# Recent Advancements in the Loading and Modification of Therapeutic Exosomes

**DOI:** 10.3389/fbioe.2020.586130

**Published:** 2020-11-11

**Authors:** Mengqiao Xu, Qianhao Yang, Xiaodong Sun, Yue Wang

**Affiliations:** ^1^Shanghai General Hospital, Shanghai, China; ^2^National Clinical Research Center for Eye Diseases, Shanghai, China; ^3^Shanghai Key Laboratory of Ocular Fundus Diseases, Shanghai, China; ^4^Shanghai Engineering Center for Visual Science and Photomedicine, Shanghai, China; ^5^Shanghai Engineering Center for Precise Diagnosis and Treatment of Eye Diseases, Shanghai, China; ^6^Department of Orthopedic Surgery, Shanghai Jiao Tong University Affiliated Sixth People’s Hospital, Shanghai, China; ^7^Department of Histology and Embryology, Second Military Medical University, Shanghai, China; ^8^Shanghai Key Lab of Cell Engineering, Shanghai, China

**Keywords:** exosome, drug delivery, engineering strategy, content loading, surface modification

## Abstract

Exosomes have a rapid development of bio-nanoparticles for drug delivery and confluent advances in next-generation diagnostics, monitoring the progression of several diseases, and accurate guidance for therapy. Based on their prominent stability, cargo-carriage properties, stable circulating capability, and favorable safety profile, exosomes have great potential to regulate cellular communication by carrying variable cargoes into specific site. However, the specific loading strategies and modification methods for engineered exosomes to enhance the targeting ability are unclear. The clinical application of exosomes is still limited. In this review, we discuss both original and modified exosomes for loading specific therapeutic molecules (proteins, nucleic acids, and small molecules) and the design strategies used to target specific cells. This review can be used as a reference for further loading and modification strategies as well as for the therapeutic applications of exosomes.

## Introduction

The past decade has witnessed the rapid development of bio-nanoparticles and confluent advances in next-generation diagnostics, monitoring the progression of several diseases, and accurate guidance for therapy (Chen G. et al., 2018; Tran et al., 2019). The research on cell-secreted extracellular vehicles (EVs), such as bio-nanoparticles, has expanded exponentially. As the main classes of EVs secreted into extracellular, exosomes play essential roles in cell-cell, cell-tissue and cross-species communication (Zhou et al., 2017), and these subjects are covered in this review.

Exosomes are specialized membranous 30–150 nm EVs released from multiple cells upon membrane fusion and harvested through body fluids or cell culture (Liew et al., 2017). Based on their prominent stability, cargo-carriage properties, stable circulating capability, and favorable safety profile, exosomes have great potential to regulate cellular communication by carrying variable cargoes into specific site (Liu and Su, 2019). Exosomes could easily across diversified barriers containing many therapeutic drugs, especially the blood/brain barriers (Bonoiu et al., 2009; De Rosa et al., 2012). The cargos in exosomes are efficiently delivered into the cytoplasm with minimal induced toxicity and include proteins, nucleic acids, and chemicals linked to the pathogenesis of many diseases (Bunggulawa et al., 2018). Moreover, the unique mechanism of targeting to the derived cells of exosomes were also highlighted, as a promising drug delivery vehicles (Park et al., 2019).

Despite the ever-deeper understanding of nanomedicine, much strategies remains to be improved before exosomes can be successfully applied for clinical applications, such as intracellular communication, immune system development, and neuron cell signaling (Alenquer and Amorim, 2015). The standard techniques for the characterization, separation, and storage need substantial development to better apply exosomes in the clinic. On the other hand, some side effects, such as transferred drug resistance or inhibited immune responses, also need to be considered to avoid deleterious outcomes. Various modified strategies of exosome generation through chemical methods or genetic engineering may overcome these barriers to improve the carrying capacity and specificity for a better therapeutic effect. This review can be used for further development of loading and modification strategies as well as for the therapeutic applications of engineered exosomes.

## The Context Loading Strategies of Exosomes for Various Substance Delivery

**FIGURE 1:**
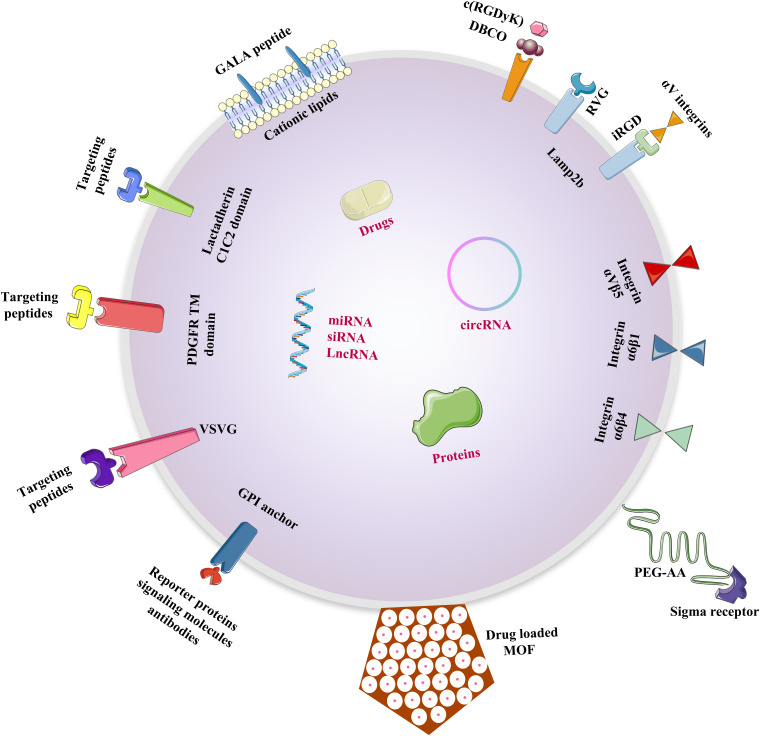
Methods of loading different cargos to specific tissues through engineered exosomes. The specific proteins, nucleic acids and small molecular drugs can be loaded into exosomes through pre-loading, post-loading and some specific strategies.

An exosome or exosome mimetics-based delivery system has desirable benefits that make it a superior choice, such as specificity to target tissues, stability, a long-circulating half-life, and biocompatibility with minimal toxic effects. Exosomes could transport different cargos to targeting tissues rapidly with high stability through their homing characteristics (Figure 1 and Table 1). As exosomes are a small and natural product, they can avoid immune responses, such as membrane fusion and phagocytosis, thus bypassing engulfment by lysosomes. The exosome can also be loaded with multiple water-soluble drugs because of its hydrophilic core.

**TABLE 1:** Different methods in existing studies of loading specific proteins, nucleic acids and small molecular drugs into engineered exosomes.

**Delivery cargo**	**Exosome source**	**Isolation methodology**	**Loading methodology**	**Therapeutic effect**	**Efficiency**	**References**
**Proteins**						
Catalase	Raw 264.7 macrophages (mouse)	Ultracentrifugation	Mixing	Preserve catalase enzymatic activity, prolong blood circulation time, and reduce immunogenicity, thereby improve drug therapeutic efficacy of many CNS disease.	4.9 (SEM ± 0.5%)	Haney et al., 2015; Zarovni et al., 2015
			Transgenic mice with loxP-tdTomato		18.5 (SEM ± 1.3%)	
			Sonication		26.1 (SEM ± 1.2%)	
			Extrusion		22.2 (SEM ± 3.1%)	
Cre recombinase	LN18	Ultracentrifugation	Transfection	identifying functional delivery of exosomes across blood-brain barrier to recipient neurons in the brain.	/	Sterzenbach et al., 2017
BDNF	Raw 264.7 macrophages (mouse)	Ultracentrifugation	Incubation	The delivery is enhanced in the presence of brain inflammation, a condition often presents in CNS diseases.	∼20%	Yuan et al., 2017
**Nucleic acid**						
*BACE1* siRNA	Dendritic cells	Ultracentrifugation	Electroporation	Specific gene knockdown after specific siRNA delivery to the brain for Alzheimer’s disease	∼20%	Alvarez-Erviti et al., 2011
*BCR-ABL* siRNA	HEK293T	Ultracentrifugation	Transfection	overcome pharmacological resistance in CML cells	/	Bellavia et al., 2017
*KRAS^*G*12*D*^* siRNA	Mouse fibroblasts	Ultracentrifugation	Electroporation	Suppression of tumor growth in pancreatic cancer	/	Kamerkar et al., 2017
MAPK siRNA	Plasma (human)	Ultracentrifugation	Electroporation	The MAPK-1 was down-regulated in monocytes and lymphocytes	/	Wahlgren et al., 2012
RAD51 and RAD52 siRNA	Malignant ascites fluid (mouse)	Ultracentrifugation	Mixing with lipofectamine	causing post-transcriptional gene silencing and massive reproductive cell death in recipient cells.	//	Shtam et al., 2013
GAPDH siRNA	Primary immature Dendritic cells	Ultracentrifugation	Electroporation	Specific gene knockdown after specific siRNA delivery to the brain for Alzheimer’s disease	10–38%	Alvarez-Erviti et al., 2011
VEGF siRNA	Dendritic cells	Ultracentrifugation	Electroporation	Suppression of tumor growth in breast cancer.	3%	Wang et al., 2017
*Let-7a* mimic	HEK293	Ultracentrifugation	Transfection	therapeutically to target EGFR-expressing cancerous tissues with nucleic acid drugs for breast cancer.	/	Ohno et al., 2013
Cas9/sgRNA	SKOV-3	ExoQuick	Electroporation	suppressing expression of poly (ADP-ribose) polymerase-1 (PARP-1), resulting in the induction of apoptosis in ovarian cancer	∼1.75%	Kim et al., 2017
miRNA	Glioblastoma cells	Ultracentrifugation	Transfection	Providing diagnostic information	/	Skog et al., 2008
	Human cord blood endothelial colony-forming cells	Ultracentrifugation		Protected kidney function and reduced kidney injury	/	Vinas et al., 2016
**Small-molecule drugs**						
Paclitaxel	Raw 264.7	ExoQuick	Sonication	Overcome MDR cancer and Reduced pulmonary metastases *in vitro* and *in vivo*	28.29 (SEM ± 1.38%)	Kim et al., 2016
			Mixing		1.4 (SEM ± 0.38%)	
			Electroporation		5.3 (SEM ± 0.48%)	
	Milk	Ultracentrifugation	Incubation	Oral	7.9 (SEM ± 1.0%)	Agrawal et al., 2017
	LNCaP and PC-3 (human)	Ultracentrifugation	Mixing	Enhanced drug cytotoxicity to prostate cancer cells	9.2 (SD ± 4.5%)	Saari et al., 2015
	Mesenchymal stromal cells	Ultracentrifugation	Incubation	Inhibited growth of human pancreatic adenocarcinoma cell	/	Pascucci et al., 2014
Doxorubicin	Immature dendritic Cells transfected with the vector expressing iRGD-Lamp2b fusion proteins	Ultracentrifugation	Electroporation	Specific drug delivery to the tumor site and inhibited tumor growth	<20%	Tian et al., 2014
	Reticulocytes	Magnetic separation	Incubation			
	LIM1215	Ultracentrifugation				
	Raw 264.7	ExoQuick	Sonication		8.0–11.0%	Lee et al., 2018
Curcumin	Mouse lymphoma cell (EL-4) and RAW 264.7 cells	Ultracentrifugation	Mixing	Enhanced anti-inflammatory activity	/	Sun et al., 2010
	Tumor cells (GL26-Luc, BV2, 3T3L1, 4T1, CT26, A20, and EL-4)			Inhibited brain inflammation and delayed brain tumor growth		Zhuang et al., 2011
Dopamine	Kunming mice blood	Ultracentrifugation	Incubation	Enhanced therapeutic effect due to brain-specific drug delivery	/	Qu et al., 2018)()

### The Engineering Strategies of Exosomes Loading With Proteins

The sufficient protein supplement is essential for the normal physiological process. For example, the deficiency of specific tumor suppressors is the leading cause of malignant tumors. Competent drug delivery vectors have great potential to accurately transporting therapeutic cargoes. The biological performance of exosomes has been extensively researched nowadays. The major hurdle has been the adequate loading of the desired protein into exosomes with minimum influence to the cell membrane and bioactivity of cargoes. Currently, several methods have been listed for exosomal loading to target specific tissues.

#### Overexpression of Cytoplasmic Proteins

The genetic engineering procedure for loading exosomes is conducted by transfection specific genes of interest into donor cells. Protein synthesis is modulated by the inserted genes, and then, proteins are secreted in exosomes through natural packing processes. After isolation and purification, engineered exosomes can be obtained (Hall et al., 2016).

Although this approach seems feasible and straightforward, there are many problems to be resolved. The proliferation and apoptosis of donor cells might be imbalanced due to overexpressed protein. A sophisticated sorting system should be used to overcome challenges of non-specific target proteins being loaded with high efficiency in exosomes.

#### Fusion Proteins Targeted to Exosomes

Several strategies have been designed to explicitly load target proteins. Di Bonito et al. have used dedicated DNA vector, which could express the fusion protein combining HPV-E7 and Nef exosome-anchoring protein. Fusion proteins that are not degraded and externally neutralized can be delivered into exosomes, which improves the specificity and loading efficiency of targeted proteins in exosomes (Manfredi et al., 2016).

#### Reduction in the Permeabilization of the Exosome Membrane

The challenges to developing catalase as a therapeutic agent were caused by the rapid metabolism of catalase and the penetration across blood-brain barrier (BBB). Batrakova and coworkers reported the new strategy to deliver catalase as the specific treatment for neuronal disease (Haney et al., 2015). After isolated from RAW 264.7 mouse macrophages, the exosomes loaded with catalase could easily transferred across multiple barriers with saponin, as a brand-new strategy reducing the permeabilization, compared with previous loading methods. The incredible neuroprotective effects could be easily achieved after intranasal administration of the exosomes (Haney et al., 2015).

#### Intrinsic Modification of the Target Protein

As a natural transporter of antigens, exosomes could efficiently carry certain vaccine for many treatments. Ubiquitin-fused target proteins were originated from human embryonic kidney (HEK 293) cells. After the protein was fused to ubiquitin, the concentration of the target protein was increased approximately ten-fold in the exosomes. Additionally, the immunoreaction of T cell might be caused by the recombinant antigen from the exosomes (Cheng and Schorey, 2016).

The ubiquitination of targeted protein has also been applied as an effective loading strategy by Sterzenbach. Cre recombinase was designed for ubiquitination, labeled with WW tag and can be easily recognized by late-domain (L-domain) pathway (Sterzenbach et al., 2017). Therefore, protein ubiquitination provides a specific signal for protein delivery and loading into exosomes. However, the degradation and dysfunction of the modified protein still need to be considered.

#### Various Mechanical Methods

Several mechanical methods, including mechanical extrusion, saponin permeabilization, incubation at room temperature, repeated freeze-thaw cycles, and sonication were verified by Haney et al. for loading of catalase to treat Parkinson’s disease (PD). The overexpressed cytoplasmic protein combined with mechanical selection such as sonication, saponin permeabilization, and mechanical extrusion were highly efficient, protecting the catalase from degradation and maintaining a sustained release. Then, the exosomes were efficiently loaded and internalized for the treatment of neuronal disease (Haney et al., 2015).

There are still some technical difficulties to be resolved. The purification of exosomes remains laborious, and the integrity and biological activity of exosomes are incompletely resolved to date. The composition of the mechanically generated exosomes might be different with cell-generated exosomes with low efficiency.

### The Engineering Strategies of Exosomes Loading With Nucleic Acids

The sufficient supplement of DNA, RNA and various nucleic acids is essential for the normal physiological process. Exosome has been widely used for the development of genetic therapy, as an effective delivery strategy for the genetic materials. Nowadays, several methods have been listed for exosomal loading to target specific tissues through the alteration of gene and protein expression.

#### Transfection With Different Reagents

After the isolation of exosomes from HeLa cells, the mixture of short interfering RNA (siRNA) AF488 and lipofectamine was incubated with exosomes at room temperature (RT) for 30 min. The exosomes loaded with siRNA were then transferred to recipient cells after being in culture for 24 h (Shtam et al., 2013; Li et al., 2018).

#### Electroporation

The concept of engineered exosomes to deliver bioactive molecules was first realized by Alvarez-Erviti et al. (2011). After loading with small interference (si) RNA, with the successful fusion of membrane protein Lamp2b of exosomes and rabies viral glycoprotein (RVG) peptide, purified exosomes from dendritic cells could target the to neuronal cells for subsequentially therapy. The RVG-linked exosomes were then injected intravenously with *GAPDH* siRNA to verify the capability and feasibility of their transport across the blood-brain barrier (BBB), as well as the effective *GAPDH* knockdown. BACE1-targeted siRNA and KRAS^*G*12*D*^ siRNA were also loaded into exosomes by electroporation for treating Alzheimer’s disease and suppressing tumor growth (Alvarez-Erviti et al., 2011; Kamerkar et al., 2017).

The abovementioned direct methods, such as loading with transfection reagents or electroporation, require repeated separation and purification. Moreover, multiple methods have been used for the purification of exosomes, including differential ultracentrifugation, size-based separation, and exosome precipitation (Heinemann and Vykoukal, 2017; Niu et al., 2017). Repeated purification and high-speed centrifugation might result in the loss of exosomes and/or a reduction in sample quality.

#### Guidance of Proteins, Peptides or Signature Sequences

New technology has also been developed based on the abovementioned disadvantages, including the RNA packaging device for mRNA delivery to exosomes and to target cells. The method is relatively efficient and does not require a high concentration of exosomes. The mRNA of catalase was readily delivered into the brain by engineered exosomes, where it significantly reduced neuroinflammation and neurotoxicity (Kojima et al., 2018). The protein L7Ae combined with C/D box were used as the RNA-packaging device (Saito et al., 2010). The gap junction protein Cx43 was also applied for the cytoplasmic transfer. With these tools, various mRNAs loaded with engineered exosomes could efficiently transfer to the target cells.

The enriched RNAs (eRNAs) has also been applied for RNA targeting in exosomes. The three motifs with conserved sequences were found in an analysis of RNAs in exosomes. Therefore, the signature sequences in these studies could be used as the loading strategy for extended therapy (Batagov et al., 2011).

#### Transfection

The protein packaging delivery methods described above could also be applied to miRNAs, which are naturally secreted into exosomes upon transfection into parental cells.

After transfected with *let-7a*, as a specific miRNA for tumor suppression, engineered exosomes were intravenously administered to suppress tumor growth by Ohno et al. (2013). Moreover, miR-124, miR-126, and HGF siRNA derived from cells of various origins were also transfected. The obtained exosomes containing these nucleic acids were verified for different therapeutic effects on cancer cells and myocardial injury (Luo et al., 2017; Wang et al., 2018; Zhang et al., 2018). However, the cytotoxicity, inefficient packaging, and poor specificity still need to be solved.

#### The Powerful Tool of CRISPR/Cas9 for Generating Exosome-Liposome Hybrids

As an effective and popular tool for genomic studies, CRISPR/Cas9 could precisely alter the DNA sequence of the target gene by a single guide RNA (sgRNA). For example, Cas9 and sgRNA targeting poly(ADP-ribose) polymerase 1 (PARP-1) have been applied for the engineered exosomes by electroporation, while the loading efficiency for such macromolecular nucleic acids is relatively lower (Lamichhane et al., 2015; Kim et al., 2017). Therefore, hybrid exosome–liposome nanovesicles were designed by Tan et al. using the pure incubation method to deliver CRISPR-Cas9-expressing vectors more efficiently. These hybrid nanoparticles were endocytosed and efficiently suppress relative gene expression in mesenchymal stem cells (MSCs) (Lin et al., 2018). The promising application of this exciting approach in gene manipulation awaits further evaluation.

In summary, despite the extensive use of electroporation, some RNAs with unique structures, such as modified miRNAs, shRNAs, mRNAs, or RNAs, are not suitable for exosomes used in clinical application. The EXOtic devices for specific mRNAs and eRNAs have significantly enhanced loading efficiency for further application.

### The Engineering Strategies of Exosomes Loading With Small-Molecule Cargo

Extensive research has demonstrated that exosomes act as vehicles to deliver chemotherapy drugs for therapeutic application. The loading methods include sonication, direct mixing, incubation, and eddy current oscillation.

Paclitaxel (PTX) is not commonly used in the clinic for solid tumors due to its exceedingly poor aqueous solubility, but it can be loaded into exosomes by multiple cycles of sonication with higher efficiency than has been achieved with other methods, such as incubation, saponin permeabilization, freezing and thawing, or extrusion (Haney et al., 2015; Kim et al., 2016). The administration of exosomal paclitaxel was further evaluated and found to exert significant antimetastatic efficacy and superior survival ability for metastatic tumor growth compared to Taxol (Kim et al., 2018). Moreover, the exosomes were also loaded with paclitaxel by the pure incubation method with increasing drug loading efficacy to form orally administered exosomal paclitaxel. Electrical perforation and ultrasonic treatment were also applied as the loading strategy with lower efficiency.

Moreover, iRGD exosomes and reticulocyte (RTC)-derived exosomes were designed for loading with doxorubicin by electroporation and moderate stirring to improve the payload efficiency to the tumor tissue (Tian et al., 2014; Qi et al., 2016). These passive loading methods are widely used for the loading of small molecule drugs; however, the degradation and loss of exosomes remains unabated in the process of purification. Moreover, the bioactivity and stability of these exosomes are significantly affected upon prolonged treatment and physicochemical properties. Therefore, the stability of exosomes, proper storage conditions and more loading strategies are worthy of study.

## Surface Modified Exosomes for Various Substance Delivery

Currently, confluent advances have been made with respect to bio-nanoparticles in terms of next-generation diagnostics, disease surveillance, and individual diagnosis and therapy. Despite ever-deeper understanding, the advances of modification of exosomes are essential for the improvement of the clinical translation.

**FIGURE 2:**
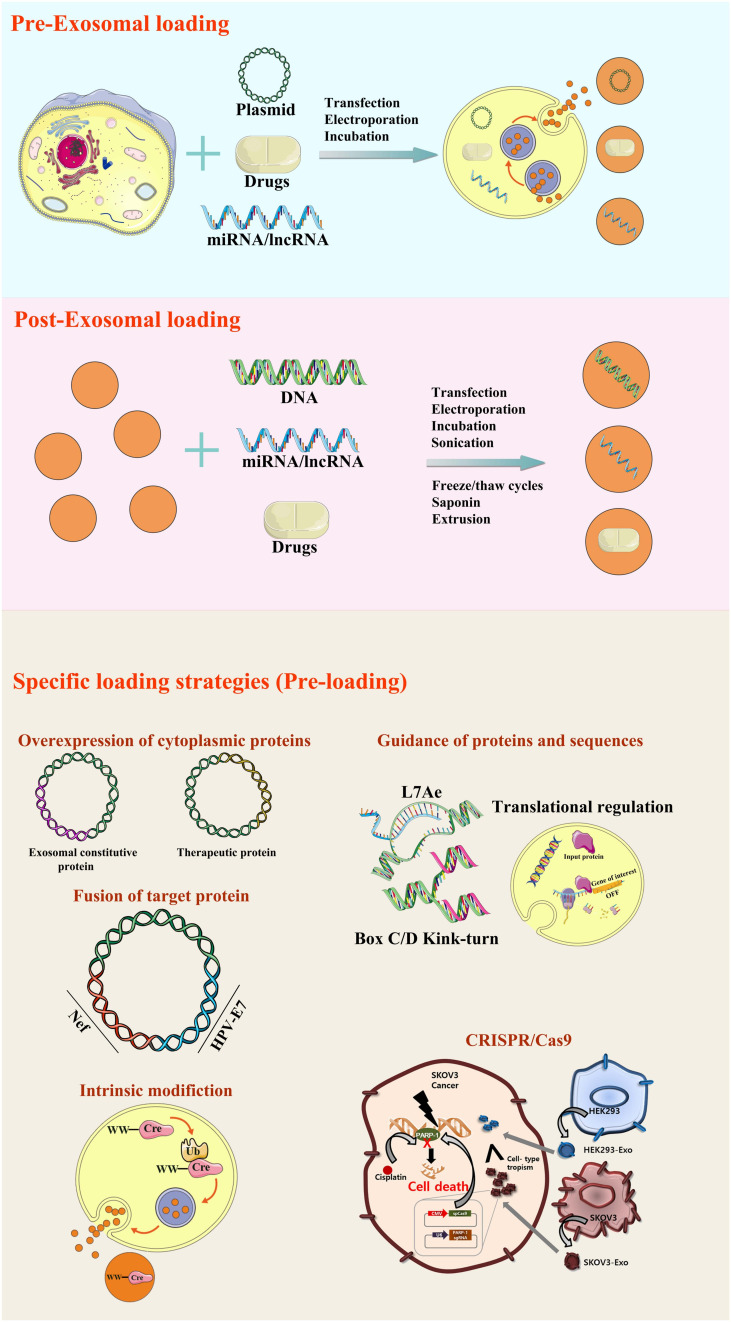
Design strategies for the modifications of exosomal surface. Multiple strategies to be done before exosomes can be successfully translated into new technologies to improve the targeting ability of donor cells and therapeutic efficacy of chemical and biomolecular drugs.

The delicate drug delivery strategy targeting to a particular tissue or a specific type of cell could avoid decentralized distribution to other tissues and preventing degradation by immune responses (Barile and Vassalli, 2017). Exosomes exhibited excellent biocompatibility and low-level long-term with natural origin compared with those of other DDSs. Therefore, modifications to the exosomal surface and components of the donor cells need to be studied for the improvement of targeting strategies (Figure 2).

### Surface-Modified Exosomes for Cerebral Ischemia Therapy

After inserting targeting proteins into donor cells, exosomes containing these targeting proteins are secreted and can be harvested and used by target antigens. Recently, the lactadherin C1C2 domain combined and Lamp2b were both used for accurate targeting.

In addition, αV integrins can be recognized explicitly by iRGD, which was loaded onto exosomes targeting tumor cells for the treatment of malignant tumors. The large amounts of exosomes from immature dendritic cells (imDCs) with an overexpressed fusion protein of iRGD and Lamp2b might be obtained by purification carried out with iRGD-targeting proteins. The imDC-exosomes loaded with doxorubicin by electroporation were specifically targeted to breast cancer cells, and they showed a direct therapeutic role in breast cancer mouse models (Tian et al., 2014).

Specific surface modification can also enhance the targeting ability of exosomes for cerebral ischemic therapy. The cyclo(Arg-Gly-Asp-DTyr-Lys) peptide [c(RGDyK)] and rabies virus glycoprotein (RVG) were used for explicit targeting to the brain. Tian et al. proposed that engineered c(RGDyK) exosomes loaded with curcumin, accumulated in considerable amounts in ischemic brain lesions following intravenous administration. Through chemical conjugation, bioorthogonal copper-free azide-alkyne cycloaddition was applied for generating mesenchymal stromal cell (MSC)-derived exosomes for integrin αvβ3 precisely located in ischemic tissue (Tian et al., 2018). Modified exosomes expressing specific RVG peptides could efficiently deliver miR-124 and opioid receptor (MOR) siRNA to a specific site in the brain, exhibiting accurate treatment for brain infarction and prevention of morphine relapse (Liu et al., 2015; Yang et al., 2017).

It is of great potential to artificially design the modification the membranes of donor cells and exosomes, thus targeting the specific tissues. Nanobodies that anchor glycosylphosphatidylinositol (GPI) on the surfaces of EVs can exhibit multiple proteins, such as reporter proteins, signaling molecules, and antibodies.

### Exosomes Combined With pH-Sensitive Fusogenic Peptides and Cationic Lipids for Cytosolic Delivery

In 2014, a repeating sequence of Glu-Ala-Leu-Ala, GALA and exosomes were combined by Nakase and Futaki for cytosolic delivery. As a cationic lipid, GALA is a pH-sensitive peptide with an amphiphilic structure and higher efficiency (Liu et al., 2015). The GALA peptide was intrinsically applied with cationic lipids to achieve the cytosolic delivery of peptides and proteins, and it also interacts with cell membranes to enable viral gene escape from acidic endosomes using a mimicking effect (Chen W. et al., 2018).

### Dual Ligand-Mediated Exosome Engineering

Dual ligand-mediated endocytosis can be applied for inducing the accumulation of exosomes at specific target sites. The permeability of the exosome membrane can be reduced to enhance the release of drugs that inhibit tumor development, enabling precise cancer therapy applications in the future.

Recent research has emphasized attempts to overcome non-small cell lung cancer (NSCLC), the predominant type of lung cancer (Liu et al., 2018), by exosome engineering strategy. The modification with polyethylene glycol-aminoethyl anisamide (PEG-AA) could acted as the dual legend to enhance the exosome circulation time and allow for the targeting of pulmonary metastatic tissues with sigma receptor overexpression. By using this modified exosome loaded with PTX and PEG-AA, the specific drug can be delivered to target cells with high loading capacity (Kim et al., 2018).

### Engineering Exosomes by Fusion With Liposomes

As a superior drug delivery vehicle, liposomes can load drugs within the aqueous compartment and concentric lipid bilayers, improving the therapeutic efficiency for pharmaceuticals, and vaccines (Bozzuto and Molinari, 2015).

Sato et al. applied the freeze-thaw method to help the fusion of liposomes with exosomal membrane. The delivery efficiency was significantly optimized because of the decreased immunogenicity of the exosome surface and increased colloidal stability with higher circulation time. The study demonstrated a new technology for engineering hybrid exosomes as bio-nanotransporters, which can be used for hydrophilic cargos to recipient cells by the specific fusion strategy (Sato et al., 2016).

### Exosome-Coated Metal-Organic Framework Nanoparticles

Metal-organic frameworks (MOFs) constitute a class of crystalline materials with diverse modularity, high crystallinity, exceptional porosity, and intriguing architectures that can be employed in gas storage, purification, and separation, as well used for catalysis and sensing applications (Zhou et al., 2018). MOFs consist of one-, two-, or three-dimensional (1D, 2D, or 3D) structural topologies as organic–inorganic hybrids with tight junction between transition-metal cations and multidentate organic linkers (Liu et al., 2016). Therefore, MOFs can be applied in nanomedicine due to sufficient loading capacity, controlled drug-release properties, and intrinsic biodegradability (Al Haydar et al., 2017).

In 2017, Illes et al. combined exosomes with MOFs to generate a smart and efficient drug carrier with an “onboard trigger.” The features of MOF NPs and exosomes were intrinsically combined, facilitating efficient and straightforward loading and retention of the cargo. Moreover, for intracellular release, the cargo was decomposed into substances mediated by endogenous exosomal release mechanisms and intrinsic biodegradability, showing great deliverability without premature leakage (Illes et al., 2017).

## Discussion

**FIGURE 3:**
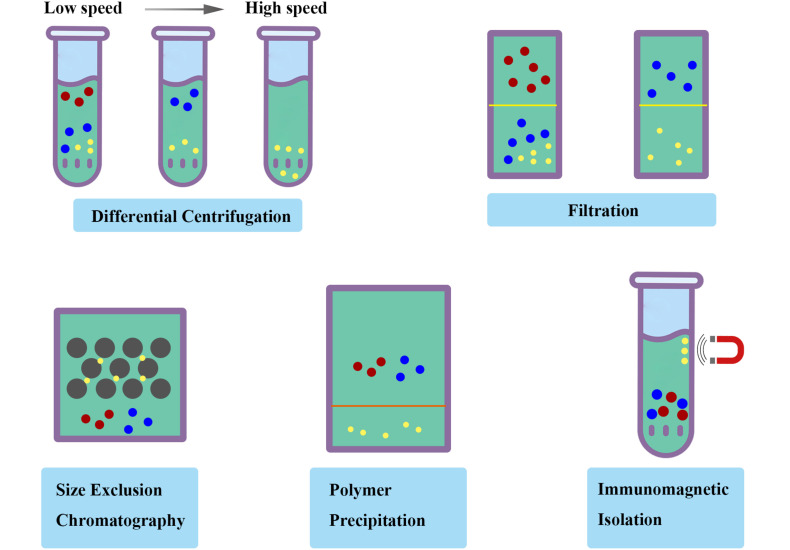
Schematic diagram of isolation methodology of exosomes. Ultracentrifugation and filtration are the most common strategies with various disadvantages. The standardized and innovative isolation protocol such as size-exclusion chromatography (SEC), polymer precipitation and immunomagnetic isolation will significantly improve the quality and consistency of exosome research.

Since exosomes were first designed for drug delivery by leveraging the ability of mRNAs and miRNAs (Valadi et al., 2007), exosomes have been widely applied for the delivery system with multiple superiority, such as specificity to target tissues, stability, a long-circulating half-life, and biocompatibility. However, several essential factors still need to be considered for further application:

(i) Homing sources. Raw 264.7 macrophages, dendritic cells, and mesenchymal stromal cells are the most common sources for the exosomes, which could significantly affect the specific content and biological performance. The specific roles of various exosomes originated from different cells are essential for further research. For example, the macrophage-derived exosomes could easily across BBB without any modifications for drug delivery. The exosomes derived from rhabdomyosarcoma (RMS) cell, osteoclast and bronchial fibroblast can be applied for specifically targeting fibroblast cells, osteoblast cells and epithelial targets. Additionally, the exosomes from malignant mesothelioma (MM) cells, metastatic cancer cells and pancreatic cancer cells (PCC) could directly targeting cancer cells for cancer treatments. It is great potential to choose appropriate homing sources for further studies.

(ii) Isolation methodology. Ultracentrifugation and filtration are the most common strategies for the isolation of exosomes. The previously reported isolation methods could not efficiently be applied in the fields of therapeutics with various disadvantages, including low yield, reduced purity, disrupted structure, and incomplete properties. Furthermore, a great challenge still existed for separating exosomes contaminated with various substances, including liposome, protein and RNA. On this issue, Möller et al. found that size-exclusion chromatography (SEC) combined with ultrafiltration was fast and efficient with highly purity and integrity for isolation approaches (Lobb et al., 2015), which is a strategy measured by the efficiency penetrating the stationary phase based on the size of different compounds. Moreover, an immunomagnetic strategy reported by Pedersen et al. could efficiently obtain ultrapure exosomes by targeting exosomal markers (Li et al., 2017). Polymer precipitation were also applied to isolate and purify polymers based on the formation of mesh-like net, with the advantage in the detection of biomarkers in vesicles (Deregibus et al., 2016; Figure 3). A standardized and innovative isolation protocol will significantly improve the quality and consistency of exosome research.

(iii) Loading strategies for engineered exosomes. Several mechanical methods, including mechanical extrusion, electroporation, saponin permeabilization, incubation at room temperature, repeated freeze-thaw cycles, and sonication were widely verified. The purification of exosomes remains laborious, and the integrity and biological activity of exosomes are incompletely resolved with some technical difficulties depending on these conventional methods. The appropriate choice and newly designed advances were essential for the loading strategy. For example, comparing the multiple loading methods for paclitaxel, such as pure incubation, mixing, electroporation, and sonication, the loading efficiency was found to be enhanced by approximately 30%, without compromised stability or functionality of the exosomal formulation (Kim et al., 2016). Moreover, exosomes loaded with magnetic nanoparticles can be targeted to external magnetic field, including the superparamagnetic nanoparticle and iron oxide. Therefore, the large-scale production of engineered exosomes can be explored and expanded with higher loading efficiency and accurate positioning.

(iv) Modification of exosomes for drug delivery. The surface modification, fusion proteins, dual legend, and fusion with liposomes have been currently applied for the method of the modification (Alvarez-Erviti et al., 2011; Ohno et al., 2013; Bellavia et al., 2017). Despite ever-deeper understanding, the advances of modification of exosomes are essential for the improvement of the clinical translation (Jia et al., 2018; Tian et al., 2018). The delicate drug delivery strategy targeting to a particular tissue or a specific type of cell could avoid decentralized distribution to other tissues and preventing degradation by immune response (Wen et al., 2016). Target T cells, cell receptors, cell adhesion molecules and antigen presentation are four main type for surface modification. The cell-penetrating peptides and T7, a transferrin receptor-building peptide were reported recently for surface modification to enhance targeting ability. Furthermore, the low level long-term accumulation in organs and tissues with concomitant low systemic toxicity is also facilitated by cellular uptake. Therefore, modifications to the exosomal surface and components of the donor cells need to be studied for the improvement of targeting strategies. It must also be clear how to convert theoretical research tinto clinical contributions with large-scale prospective studies.

## Conclusion

The past decade has witnessed confluent advances in the knowledge of exosomes for next-generation diagnostics, disease surveillance, and individual diagnosis and therapy, which was widely applied for early diagnosis and delivery system with higher efficacy. Further advanced and improvements for the drug loading strategies and modification methods will be favor of the clinical translation in the future.

## Author Contributions

YW and XS proposed the conception for the review. MX and QY wrote the manuscript and prepared the figures and table. YW and MX gave critical discussions and revisions on manuscript. All authors read and approved the final manuscript.

## Conflict of Interest

The authors declare that the research was conducted in the absence of any commercial or financial relationships that could be construed as a potential conflict of interest.
